# Deep Adaptive Ensemble Filter for Non-Intrusive Residential Load Monitoring

**DOI:** 10.3390/s23041992

**Published:** 2023-02-10

**Authors:** Nasrin Kianpoor, Bjarte Hoff, Trond Østrem

**Affiliations:** Department of Electrical Engineering, UiT—The Arctic University of Norway, 8514 Narvik, Norway

**Keywords:** load disaggregation, non-intrusive load monitoring, flexible load, signal processing, deep learning

## Abstract

Identifying flexible loads, such as a heat pump, has an essential role in a home energy management system. In this study, an adaptive ensemble filtering framework integrated with long short-term memory (LSTM) is proposed for identifying flexible loads. The proposed framework, called AEFLSTM, takes advantage of filtering techniques and the representational power of LSTM for load disaggregation by filtering noise from the total power and learning the long-term dependencies of flexible loads. Furthermore, the proposed framework is adaptive and searches ensemble filtering techniques, including discrete wavelet transform, low-pass filter, and seasonality decomposition, to find the best filtering method for disaggregating different flexible loads (e.g., heat pumps). Experimental results are presented for estimating the electricity consumption of a heat pump, a refrigerator, and a dishwasher from the total power of a residential house in British Columbia (a publicly available use case). The results show that AEFLSTM can reduce the loss error (mean absolute error) by 57.4%, 44%, and 55.5% for estimating the power consumption of the heat pump, refrigerator, and dishwasher, respectively, compared to the stand-alone LSTM model. The proposed approach is used for another dataset containing measurements of an electric vehicle to further support the validity of the method. AEFLSTM is able to improve the result for disaggregating an electric vehicle by 22.5%.

## 1. Introduction

The demand for power and energy is increasing due to the growing electrification of society. This creates severe problems of power and energy shortages. The residential sector is one of the sectors that has a large share of the end use of energy after industry and transport. In 2019, energy consumption in the household sector represented 26.3% of the final consumption in the EU, which is a significant amount [1]. This amount can be reduced using demand-side management strategies [2].

In a traditional power system, loads cannot be controlled, leading to higher consumption and subsequently higher pressure on the grid. However, it is possible to control the flexible loads in the residential sector using HEMS [3]. Flexible loads are used to reduce electricity consumption in peak load periods and shift it to off-peak load periods or times when the electricity price is low. Heat pumps, water heaters, floor heating cables, and electric vehicles are some examples of flexible loads in a residential building [4]. A balance between energy consumption and production can be provided using HEMS technologies by participating in flexible loads in different DR programs, considering consumer comfort preferences [5]. Providing feedback to the consumers gives them a better insight into managing their electricity consumption through a suitable DR program [6].

The first step in controlling flexible loads is to monitor them. The process of identifying and obtaining the load signatures in a power system is load monitoring [7]. Load signature is the intrinsic consumption pattern of each individual appliance. Each electrical appliance has its own unique features when it is in operation.

**Figure 1:**
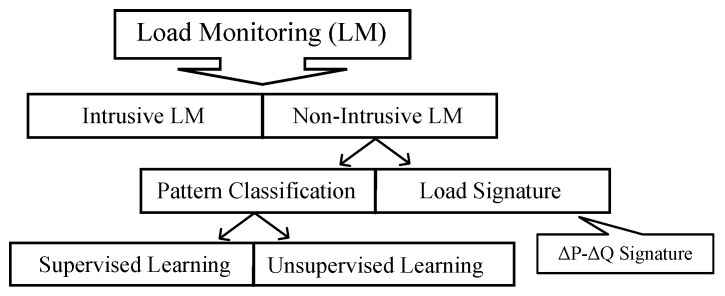
Different load monitoring methods.

Generally, there are two main methods for load monitoring, the first is intrusive load monitoring and the second is non-intrusive load monitoring. ILM is a traditional method for load monitoring. In this method, each device has its own measuring sensor to record the power consumption of the device with a specific time resolution. Even though ILM is a precise method and the signatures of individual appliances can be obtained using direct measurements, there are drawbacks to this method, because a sensor needs to be installed for each appliance in order to acquire the power consumption data of that specific device. Therefore, it is a cumbersome process, and the large number of home appliances in each house leads to high maintenance and hardware costs [8]. In order to solve this problem, Hart proposed a non-intrusive load monitoring concept for the first time in the 1980s [9]. In simple terms, the process of extracting the load signature of individual appliances from the total power using computational methods is NILM or load disaggregation. In NILM, only one set of measurement sensors is required at the power entrance to record the aggregate power data. Based on the data that are used to solve the problem, it can be categorized into supervised and unsupervised learning methods. In supervised learning, data have a label and there is information about different appliances to train the network, while unsupervised learning data are unlabelled [10]. Depending on the requirements, supervised learning can be defined as a regression or classification problem. Different load monitoring methods are categorized in Figure 1. In this paper, a supervised learning method is used for load monitoring.

### 1.1. Literature Review

There are two main approaches to NILM [11]: state-based and event-based methods. The event-based approaches use edge detection techniques to identify the events in the signal. The total power consumption in a home is constantly changing, and new events (ON/OFF of one or more appliances and changes in the states of the appliances) can be identified based on the edges. Load signatures of different appliances are extracted based on the intrinsic features of each device. Different classification methods including kNN [12], Decision Trees [13], HMM, SVM [14] and Naive Bayes have been employed. The similarities between appliance signatures and high measurement noise limit the performance of the event-based method. In order to improve the performance of event-based NILM, a graph signal processing algorithm was studied by Zhao et al. [15].

The state-based method represents each operation of the appliance using a state machine with distinct state transitions according to the usage pattern of appliances. When the states of appliances change or appliances turn ON/OFF, they have different edges and each of them has a different probability distribution that fits a specific appliance. The HMM and its different extensions are examples of state-based NILM approaches [16,17,18,19,20]. In the state-based method, prior values of appliances through a long period of training are required, which is a limitation of this method. High computational complexity is another drawback of the state-based method [21,22]. There is some research that has tried to solve NILM using mixed integer programming. In recent years, Wittmann et al. have [23] considered the NILM problem as a mixed integer linear programming problem and achieved high accuracy. A two-stage optimization-based method based on mixed integer nonlinear programming was presented for load disaggregation in [24].

Recently, deep learning methods have received attention in different fields of study such as image processing [25] and speech recognition [26]. Powerful computers and datasets in different fields are the main reasons for increasing the usage of deep learning methods. Due to the extensive installation of smart meters in recent years, energy consumption data in the household sector are now accessible. For this reason, different deep learning methods such as CNN [27,28,29,30,31] and RNN [1] have been widely used in the energy disaggregation problem. In [27], three different deep neural network methods were used for energy disaggregation and seven metrics were used to check the performance of these algorithms on five different appliances. A casual CNN network for load disaggregation on low-frequency data was presented in [32], in which it was concluded that using all four components (current, active power, reactive power, and apparent power) leads to higher performance. In [33], a sequence-to-point learning CNN architecture was proposed, where the sliding window was used for input data to manage long-term time series. Athanasiadis et al. [34] proposed a scalable real-time event-based load disaggregation algorithm using the CNN network. The proposed algorithm included three parts: an event detection algorithm, a CNN classifier, and a power estimation model.

The LSTM model has also been used in the application of NILM. In Ref. [35], an energy disaggregation method based on the LSTM-RNN model was proposed. A bidirectional LSTM model was used in [36] for energy disaggregation. A U-net architecture for one-dimensional data for power estimation and multi-task-multi-appliance state detection was proposed by Faustine et al. [37]. In [38], a subtask-gated network was proposed, in which the main regression network was combined with an on/off classification subtask network.

Although some research has been undertaken on load disaggregation with different deep learning methods, few of them have considered the impact of data preprocessing methods on the proposed models, and most of the proposed methods are considered as a classification problem that detects only switch on/off events.

### 1.2. Our Contribution

This paper proposes a new adaptive load disaggregation framework (AEFLSTM), powered by the strength of signal processing methods and LSTM. The AEFLSTM is an adaptive framework that can be used for load disaggregation in residential houses by searching among an ensemble of signal processing techniques to improve the LSTM performance for disaggregating that specific load. Signal processing methods are strong tools for filtering frequencies that might not be useful for flexible load disaggregation. The LSTM is also a strong technique to learn the temporal dependencies of flexible loads and address nonlinearities and uncertainties. To the best of the authors’ knowledge, AEFLSTM is the first attempt at flexible load disaggregation which tries to find the best signal processing technique for LSTM to improve accuracy. The contributions of this paper are summarized as follows:

- In terms of methodology:
  - An adaptive disaggregation framework powered by the signal processing technique and LSTM is developed that is applicable to any load disaggregation problem;
  - The proposed AEFLSTM is a holistic algorithm that can easily be generalized to have more signal-processing techniques.
- From an application point of view, the proposed framework AEFLSTM is used to disaggregate a heat pump and a refrigerator from the total power of a residential building in British Columbia. The accuracy of load disaggregation is significantly improved by using the proposed AEFLSTM framework based on actual data from the use case.

The paper is organized as follows: The data that are used in this study are explained in Section 2. The methodology, including signal processing and deep learning methods, is presented in Section 3. Section 4 shows the results of the experimental evaluation of the proposed methods. Finally, the conclusion of the paper is given in Section 5.

## 2. Use Case

**Figure 2:**
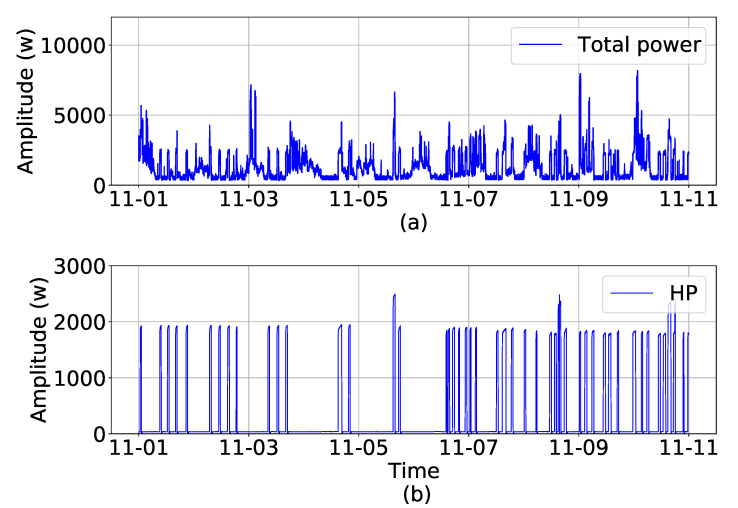
Ten days data of (**a**) total power, (**b**) heat pump from 1 November 2012 to 11 November 2012.

In this paper, data from a residential building in British Columbia, Canada are used. The dataset, named AMPDs, is an open-source dataset and is available from [39]. It contains different measured factors, such as voltage, current, frequency, real power, reactive power, etc., for different loads in the house such as a the heat pump, refrigerator, dishwasher, washing machine, and other common loads. The main focus of this paper is to disaggregate the major loads from the total power to use them as flexible loads in future research. Therefore, the focus of this study is on the disaggregation of heat pumps, which are the major electricity consumer in AMPDs data. Hence, in Figure 2, the total power of the house and heat pump power consumption is plotted. Moreover, their statistical metrics, including the mean value, standard deviation, and minimum and maximum values of the total power and heat pump power consumption, are presented in Table 1. The metrics in Table 1 show that the heat pump has a large share of electricity consumption in the AMPDs data. Therefore, it will be valuable if controlled as a flexible load.

## 3. Methodology

**Figure 3:**
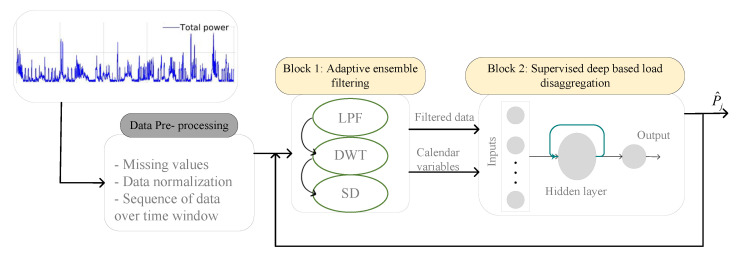
The proposed framework for load disaggregation.

In this paper, a deep adaptive ensemble filter based on various signal processing tools integrated with an LSTM is developed for flexible load disaggregation. The overall schematic of the proposed framework AEFLSTM is presented in Figure 3. As is shown, the proposed framework has two main modules, and it should be mentioned that there is a data preprocessing step before applying the main signal to the first module. The first module is an adaptive ensemble filtering block that consists of three famous signal processing methods. The output of this module is a clean total power signal in which irrelevant frequencies are filtered (e.g., noise or high frequencies), depending on the main frequencies of the selected flexible load that need to be disaggregated from the total power. Then, the filtered signal and calendar variables are applied to the second module, which is a supervised deep-based load disaggregation block, to enhance the learning ability of the LSTM for disaggregating the flexible load from the filtered signal. There is feedback from the output of the second module to the input of the first module in order to find the best signal processing method which has the best performance for load disaggregation. In other words, all the available signal processing methods in the first modules are tested by the algorithm, and the one with the best performance is selected. In the following subsections, each module as well as the data preprocessing step is explained in detail.

Consider a house with *N* different household appliances. If P(t) is the total power that is taken from the smart meter at the power entrance of the house and $P_{j}\left(t\right)$ is the power consumption of *j*-th appliance (1≤j≤N), then the total power is [13]:

(1) $$P\left(t\right)=\sum_{j=1}^{N}P_{j}\left(t\right)+e\left(t\right)$$

where e(t) is the noise. The main goal of the problem in Equation (1) is to estimate $P_{j}\left(t\right)$ with a given P(t).

### 3.1. Data Cleaning

The first step before training any machine learning algorithm is data cleaning, which can improve data quality and consistency. Data cleaning can include the following steps such as finding missing values, interpolating or imputing missing values, detecting outlier data, standardization, or normalization. In this work, fortunately, no missing values or outliers were found in the AMPDs dataset. Moreover, the data are standardized as follows [40]:

(2) $$x_{new}=\frac{x-\bar{x}}{\sigma }$$

where $x_{new}$, *x*, and $\bar{x}$ are the normalized value, real value, and mean of real values, respectively. In addition, σ is the standard deviation of the real data. In this formula, all the new values are centered around a mean value with unit variance. Generally, there is no specific rule for selecting the normalization or standardization methods and it is highly dependent on the problem [41]. In this work, standardization was selected heuristically.

### 3.2. Adaptive Ensemble Filtering

After performing pre-processing on the data, the normalized data is applied to the adaptive ensemble filtering block. This block consists of three signal-processing methods. Each time series can be broken into two main components: systematic and non-systematic. Systematic parts can be described and modeled due to their recurrence and consistency. Non-systematic parts of the time series that are known as noise cannot be modeled because they are random variations in the time series. Therefore, the model accuracy can be increased by removing the non-systematic components of the time series. In this paper, low pass filtering, discrete wavelet transform, and seasonal decomposition are applied to remove the non-systematic part of the time series. Here, each of them is briefly described.

#### 3.2.1. Low-Pass Filter

**Figure 4:**
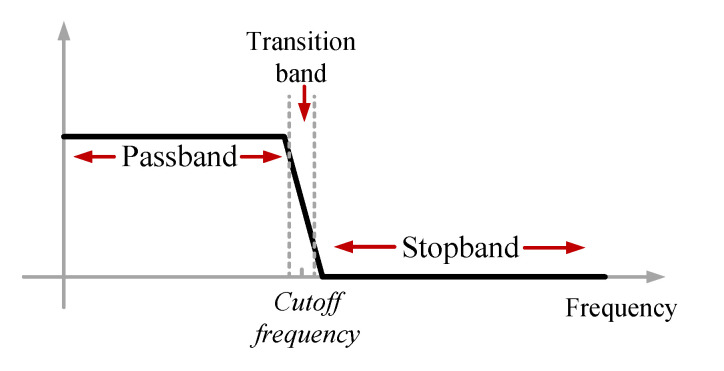
A low pass filter in the frequency domain [42].

A low-pass filter passes a signal with a frequency lower than a certain cut-off frequency. In the other words, the main aim of low-pass filtering is to remove the components above the cut-off frequency. A fast Fourier transform can be used to determine the value of the cut-off frequency because the FFT can find the frequencies, amplitudes, and noise components of the signal. The cut-off frequency value can be set manually as well. A low pass filtering in the frequency domain is shown in Figure 4. The filter passes the signal in the passband and attenuates the signal in the stopband. The cut-off frequency is in the transition band.

#### 3.2.2. Discrete Wavelet Transform

The second method that is used for denoising the time series is the discrete wavelet transform [43]. In practice, DWT is used as a filter bank that can deconstruct a signal into the low pass (approximation) and high pass (detail) coefficients, which is called signal decomposition. If the signal is reconstructed again using detail and approximation coefficients, the output will be the original signal. However, if the detail coefficients that are representative of the high-frequency part of the signal are left out in the reconstructing process, then the output signal is the original signal that is filtered out. The DWT of a time series signal *x*[*n*] is as follows:

(3) $$W_{x}\left(a,b\right)=\sum_{n}\frac{1}{\sqrt{a}}x\left[n\right]\psi^{*}\left(\frac{n-b}{a}\right)$$

where the parameter *a* sets the scale or dilation of the wavelet; it decides how squashed or stretched a wavelet is. Increasing the value of *a* will stretch the wavelet and decreasing its value will squash the wavelet. The location of the wavelet is defined by parameter *b*. Increasing the value of *b* moves the wavelet to the right and decreasing its value shifts the wavelet to the left. $\psi^{*}$ is the complex conjugate of the mother wavelet, ψ. To define DWT, the following assumptions are considered:

(4) $$\left\{\begin{array}{c} a=2^{j}\\ b=k2^{j} \end{array}\right.,\;\;\;\;\;\;\;\;\;\;j=0,1,2,\cdots \;$$

where *k* is an integer. The wavelet function is stretched in the time domain and squashed in the frequency domain by a factor of two if the index *j* increases by one.

By substituting (4) into (3), the new equation for the DWT is as follows:

(5) $$W_{x}\left(j,k\right)=\sum_{n}\frac{1}{\sqrt{2^{j}}}x\left[n\right]\psi^{*}\left(2^{-j}n-k\right).$$

**Figure 5:**
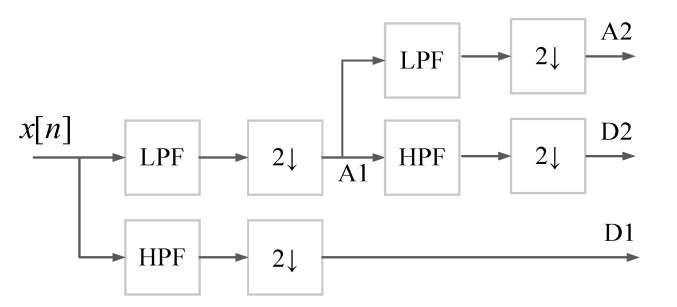
The structure of DWT decomposition model [44].

The structure of the DWT decomposition model is depicted in Figure 5.

#### 3.2.3. Seasonal-Trend Decomposition Method

The last method that has been used for denoising the signal is the additive decomposition method. Earlier in this section, it was explained that each time series has two components: a systematic part and a non-systematic part. The systematic component includes trend and seasonality. The trend component shows the long-term change (increasing or decreasing) in the time series, and the seasonality component indicates periodic cycles in the data. Therefore, a time series is a function of trend, seasonality, and noise. The equation can be written as follows [45]:

(6) $$Y_{t}=f\left(T_{t},S_{t},e_{t}\right)$$

where $Y_{t}$ is a time series, $T_{t}$ is the trend, $S_{t}$ is the seasonality, and $e_{t}$ is the noise. In an additive decomposition model, it is assumed that the time series is a combination of all components. The equation of the additive decomposition model is as follows:

(7) $$Y_{t}=T_{t}+S_{t}+e_{t}.$$

### 3.3. Supervised Deep-Based Load Disaggregation

**Figure 6:**
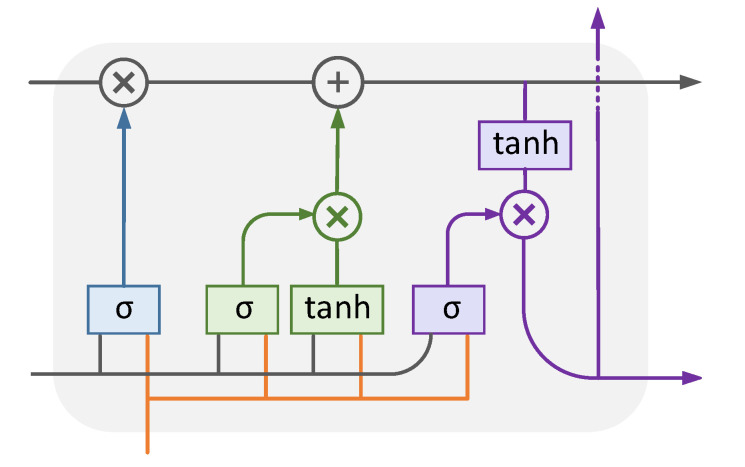
The structure of the LSTM cell.

The output of the first module and calendar variables are applied to the second module, which is a supervised deep-based load disaggregation block. In this module, the LSTM network, which is a deep learning method, is used for load disaggregation. An LSTM model is composed of different cells that are responsible for remembering information. Each cell includes three parts: the forget gate, the input gate, and the output gate. The information that is less important or is no longer needed for the LSTM is removed through the forget gate. It optimizes the performance of the network. The forget gate has two inputs, $x_{t}$ is the input at time *t* and $h_{t}-1$ is the hidden state from the previous cell. $f_{t}$ is the output of the forget gate. It is a vector with values 0 and 1; a 0 output for a specific value implies forgetting the information related to that value, whereas 1 implies that the information is remembered. The input gate adds new information to the cell. It creates a vector containing all possible values that can be added to the cell. The vector is filtered and scaled in the range of −1 to 1 by the tanh function to keep only important information. The output gate chooses useful information from the current cell. It sends them out as the hidden state for the next cell and as an output for the current cell. The structure of the LSTM cell is shown in Figure 6.

## 4. Experimental Results

To evaluate the proposed AEFLSTM model for load disaggregation, the AMPDs dataset was used. The resolution of the data is one minute, which is low frequency; therefore, it has less computational complexity than high-frequency data. The details of the dataset are explained in Section 2. The experiments were implemented in “Google Colab” using Numpy, Pandas, Keras, and sklearn libraries.

In this study, the adaptive ensemble filtering module (Block 1, shown in Figure 3) searches between different denoising methods in the block and at the end compares the result and chooses the best one for load disaggregation. The results were evaluated based on the mean absolute error (MAE) and root mean square error (RMSE), which are commonly used to evaluate the energy disaggregation problem. The formulas of the metrics are as follows:

(8) $$MAE=\frac{1}{n}\sum_{i=1}^{n}|y_{i}-{\hat{y}}_{i}|$$

(9) $$RMSE=\frac{1}{n}\sum_{i=1}^{n}{\left(y_{i}-{\hat{y}}_{i}\right)}^{2}.$$

**Figure 7:**
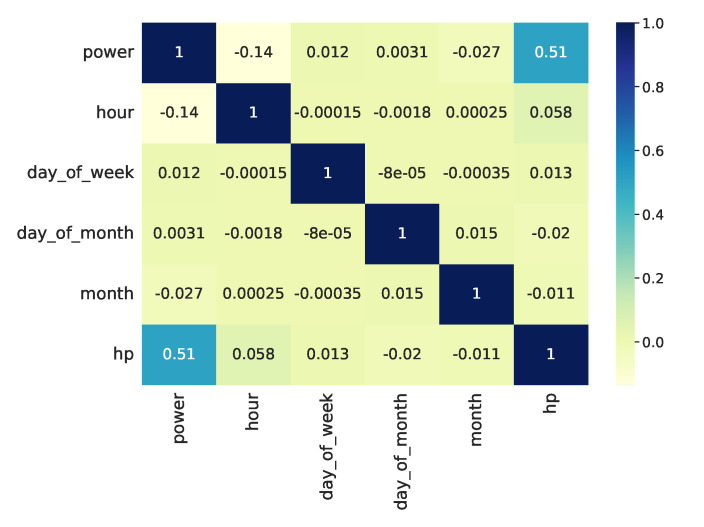
The correlation between total power and heat pump power consumption with calendar variables.

The household load consumption is highly dependent on time. Hence, it is essential to discover the correlation between power consumption and calendar variables including hours, weekdays, and months. At this stage, some analyses have been performed to show these dependencies. In Figure 7, the correlation heat map between total power and power consumption of the heat pump with calendar variables including hour, day of week, day of month, and month is shown. To calculate the pairwise correlation of columns, the corr() function in the pandas library was used, in which the Pearson method (standard correlation coefficient) was selected for the correlation function. The heat map was plotted using the seaborn library in python. As it can be seen in Figure 7, the heat pump power consumption has the highest correlation with the total power and among the calendar variables, it has the highest correlation with hour.

Before implementing AEFLSTM for load disaggregation, a stand-alone LSTM model was compared with the state-of-the-art models, including linear regression and decision tree regression, in order to determine which of them are more suitable for the application of load disaggregation. The total power consumption, history of the appliance, and calendar variables including hour, day of week, and day of month were used to train the LSTM network. The parameters of the LSTM model, including the number of training epochs, batch size, and the number of nodes to use in the hidden layer, were chosen using a grid search. The final LSTM network configuration is shown in Table 2.

The period from 1 November 2012 to 30 November 2012 of the aggregated power consumption data of the AMPDs dataset was selected for training, validation, and testing of the LSTM model. A total of 30% of the data was considered for validation and one day of the data was used for testing the model.

The input layer of the LSTM network receives the data in a three-dimensional format with a moving time window. Here, a time step of 360 min is considered, which means data are segmented into different time windows with a duration of 360.

**Figure 8:**
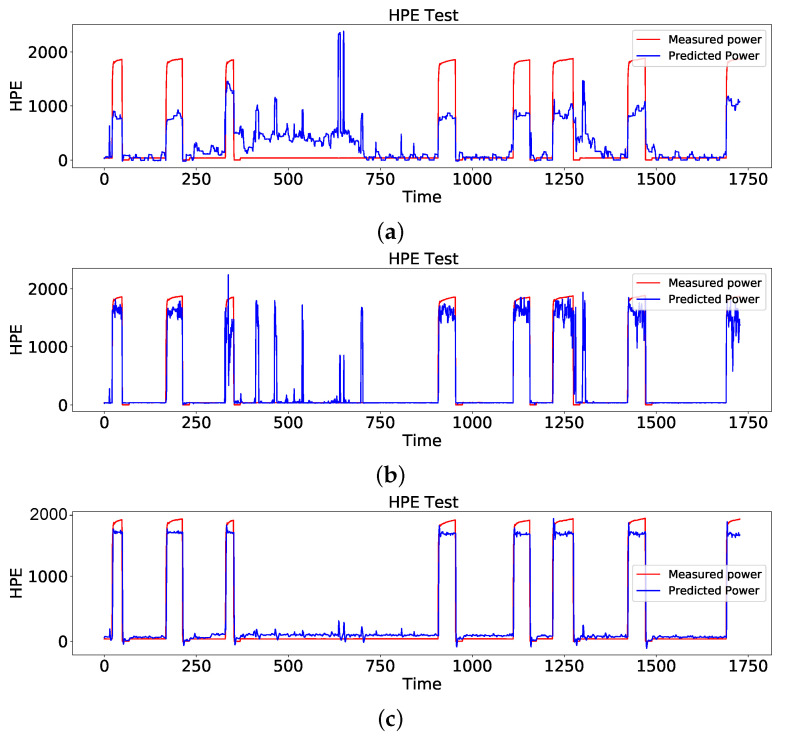
Comparison of the estimated power consumption of the heat pump with ground truth using (**a**) LR, (**b**) DTR, and (**c**) LSTM.

After network configuration, LR, DTR, and LSTM were used to disaggregate the heat pump power consumption from the aggregated total power. A numerical comparison of all the methods is presented in Table 3. As presented in Table 3, the LSTM model outperforms the other models, as the MAE error was significantly reduced from 319.35 (*W*) and 97.01 (*W*) to 90.2 (*W*). Although the LR and DTR models could detect the switch on/off events of the heat pump, they could not correctly identify the peak of the load. Another problem of these two models is that they cannot correctly distinguish the load cycle (heat pump time period) and show the device as on while it is off in reality based on measured data. For the sake of a better understanding, the DR, DTR, and LSTM performances are presented in Figure 8.

The details of different filtering methods used in the adaptive ensemble filtering block in Figure 3 are presented separately in the following subsections.

### 4.1. Case 1: LPF

**Figure 9:**
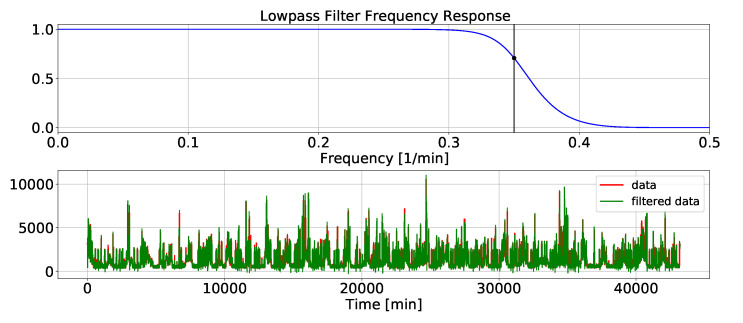
LPF smoothing.

In this section, the specification of the LPF, which is one of the filtering methods in the adaptive ensemble filtering block, is explained. A signal processing toolbox called “scipy.signal” was used. The value of the desired cut-off frequency of the filter is an important factor to design a proper LPF. Improper selection of this parameter may change the nature of the original signal. For this reason, the signal was first transferred to the frequency domain using a fast Fourier transform. The frequencies and amplitudes of the signal vs. the noise components can be identified based on the signal response in the frequency domain. A range of frequencies outside the principle frequency was selected and, based on trial and error, the number 0.35 was considered as the desired cut-off frequency. The LPF frequency response and the aggregate power consumption signal and its LPF smoothed version are depicted in Figure 9.

### 4.2. Case 2: DWT

**Figure 10:**
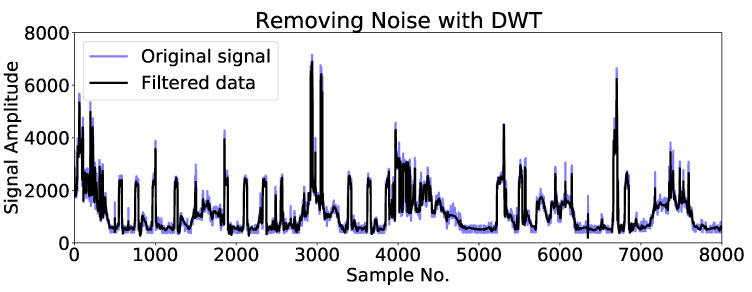
Ten-day-data of aggregate power consumption signal and filtered data using DWT smoothing.

In this section, the DWT specification used in the adaptive ensemble filtering block is explained. DWT was used to remove the noise in which the signal is deconstructed into the detail and approximation coefficients. Here “PyWavelets” (an open-source wavelet transform software for python) was applied for signal decomposition. In the simulation, one family of wavelets, called “Daubechies”, was considered. The detail coefficients were filtered out using “pywt.threshold”, which removes coefficients above a certain threshold. The aggregate power consumption signal and its DWT smoothed version are shown in Figure 10.

### 4.3. Case 3: SD

**Figure 11:**
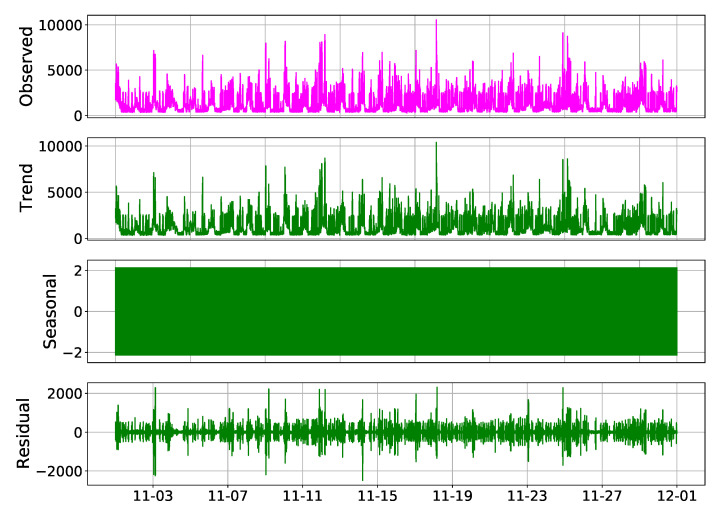
The main signal and its decomposed components.

The last method used for filtering the signal was the seasonal decompose method. In order to implement the SD model, a times series analysis toolbox named “statsmodels.tsa” was utilized. The total power consumption signal was decomposed into different components including trend, seasonality, and residual components using the “seasonal_decompose” function in the “tatsmodels.tsa” toolbox. The main signal and its components are plotted in Figure 11. As it can be seen from Figure 11, the signal does not show seasonality; therefore, the trend is considered as the extracted feature and the residual is filtered out.

**Figure 12:**
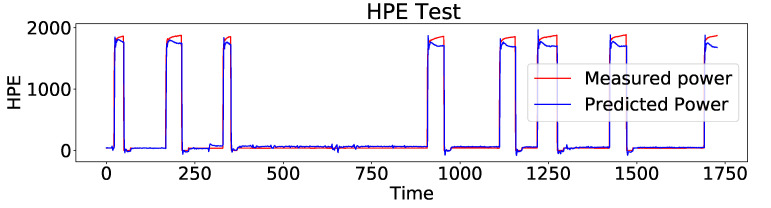
A comparison of the estimated power consumption of the heat pump with ground truth using AEFLSTM.

In the following, AEFLSTM is implemented using the configuration and parameters mentioned in Section 4.1, Section 4.2 and Section 4.3 and Table 2 for the first and second modules, respectively, to disaggregate the heat pump power consumption from the total power. The AEFLSTM algorithm searches among all the available signal processing methods in the first module and the one with the best performance is selected. The AEFLSTM model shows, DWT-LSTM outperforms the others while disaggregating the heat pump power consumption. The output of the AEFLSTM model, which is the estimated power consumption of the heat pump, is compared with the ground truth in Figure 12. Finally, the performances of all the filtering methods are compared based on the MAE and RMSE metrics in Table 4. As can be seen from the comparison, all the methods have improved the model, and their performances are better than a stand-alone LSTM network. Among them all, DWT-LSTM outperforms with a slight difference from SD-LSTM; therefore, it was chosen as the output of the AEFLSTM model.

The main focus of this paper is to disaggregate the sizeable and major loads to use them as flexible loads in future work. However, in this part, the proposed AEFLSTM method is implemented to estimate the signatures of the refrigerator and the dishwasher of the AMPDs dataset, to show the performance of the proposed methodology on other appliances as well. First, a stand-alone LSTM model, a DTR model, and an LR model were used to disaggregate the power consumption of the appliances from the total power consumption. Again, the LSTM model outperforms the two other methods for both appliances. For the refrigerator, LR failed to identify on/off events. DTR could detect a few events but was not able to identify the peak of the load. However, for the dishwasher, both methods (LR and DTR) failed to identify on/off events. A numerical comparison of these different methods is presented in Table 5.

**Figure 13:**
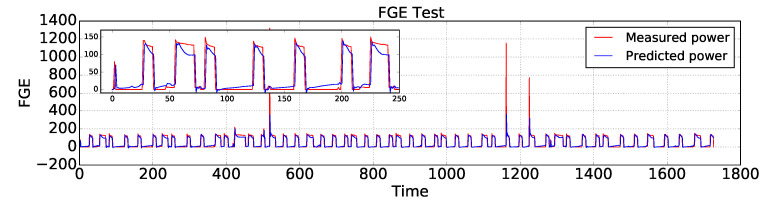
A comparison of the estimated power consumption of the refrigerator with ground truth using AEFLSTM.

**Figure 14:**
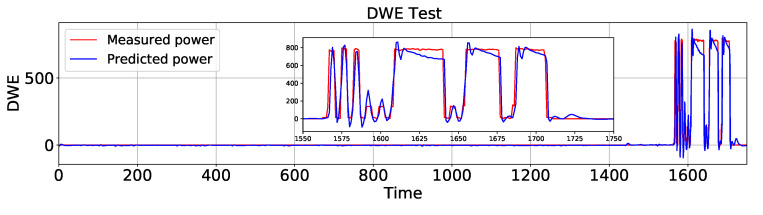
A comparison of the estimated power consumption of the dishwasher with ground truth using AEFLSTM.

In the following, AEFLSTM was implemented using the configuration mentioned in Section 4.1, Section 4.2 and Section 4.3 and Table 2 for both modules. This time, as in the previous case for the heat pump, the AEFLSTM chose the DWT-LSTM method as the best result for estimating the power consumption of the refrigerator and it chose LPF-LSTM for disaggregating the signature of the dishwasher (slightly better than DWT-LSTM) from the total power. A comparison of the estimated power consumption using AEFLSTM with ground truth data for the refrigerator and the dishwasher is shown in Figure 13 and Figure 14, respectively. The zoomed-in versions of both figures show how accurately the estimated power consumption tracks the measured data. The numerical results of both case studies are presented in Table 5.

**Figure 15:**
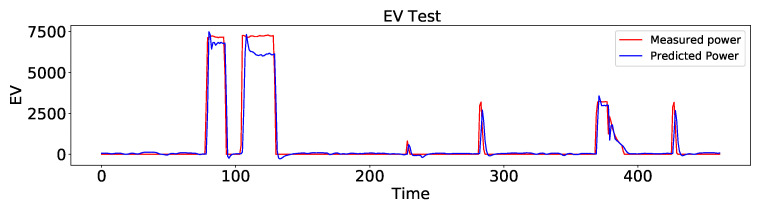
A comparison of the estimated power consumption of the elctric vehicle with ground truth using AEFLSTM.

Here, the validation phase is expanded by including another dataset to further support the validity of the proposed approach. For this reason, a dataset from a residential building in the arctic region of northern Norway is considered. The dataset contains the measurements of the electricity consumption of the main circuit and different appliances, where electric vehicles are one the major loads, consuming a large part of the total power. Therefore, the AEFLSTM approach was used to estimate the power consumption of the electric vehicle from the total power. However, before implementing the AEFLSTM, other methods, including LR, DTR, and stand-alone LSTM, were used to estimate the power consumption of electric vehicles from the total power, in which LSTM performed better. The performance metrics of the difference between real and estimated data for electric vehicles are presented in Table 5. A comparison of the proposed method with ground truth for electric vehicles is depicted in Figure 15.

## 5. Conclusions

This paper presented a new deep adaptive ensemble filter for non-intrusive residential load monitoring. This method is used for non-intrusive residential load monitoring. The proposed AEFLSTM framework searches among ensemble filtering methods in the first module to find the best method for load disaggregation application. Experimental results are presented for disaggregating a heat pump, refrigerator, and dishwasher from the AMPDs dataset. The performance of the stand-alone LSTM is compared with DTR and LR models to determine which is more suitable for the load disaggregation problem. The results show that the LSTM model outperforms in disaggregating the heat pump, refrigerator, and dishwasher. AEFLSTM is implemented to estimate the power consumption of the appliances. AEFLSTM selects DWT-LSTM as the more accurate method for disaggregating the heat pump and refrigerator signatures from the total power and LPF-LSTM for disaggregating the signature of the dishwasher. The results show that AEFLSTM can reduce the loss error (mean absolute error) for the heat pump, refrigerator, and dishwasher by 57.4%, 44%, and 55.5%, respectively, compared to the stand-alone LSTM model. Finally, another dataset containing data on electric vehicle power consumption is considered to further support the validity of the proposed approach. AEFLSTM is able to improve the result of estimating the electricity consumption of the electric vehicle by 22.5%.

Research on NILM has led to a detailed understanding of the energy consumption of home appliances. Appliances used for heating, cooling, ventilation, washing, and drying can be considered as flexible loads where their utilization may be reduced or changed during peak load periods, considering user comfort. Future work will investigate the possibility of deploying a home energy management system for appliance flexibility using NILM.

## Figures and Tables

**Table 1:** The statistic metrics of total power and heat pump.

	Mean (W)	STD (W)	Min. (W)	Max. (W)
Total power	1396.7	1132.4	269.0	10,542.0
Heat pump	407.2	737.7	0.0	3030.0

**Table 2:** The LSTM network configuration.

LSTM Parameters				
	First layer		Second layer	
Nodes			100	50
Dropout rate			0.2	0.2
Return sequence			True	False
Activation function			relu	relu
**Optimizer**	**loss**	**epotchs**	**batch_size**	**validation_split**
Adam	MSE	15	30	0.3

**Table 3:** HPE with different methods.

HPE	MAE (W)	RMSE (W)
LR	319.35	500.9
DTR	97.01	268.6
LSTM	90.2	208.68

**Table 4:** HPE with different methods.

HPE	MAE (W)	RMSE (W)
LSTM	90.2	208.68
DWT-LSTM	38.4	148.75
LPF-LSTM	51.5	157.1
SD-LSTM	39.7	153.2

**Table 5:** Performance metrics (MAE and RMSE) of the difference between real and estimated data for different appliances.

	FGE		DWE		EV	
	MAE (W)	RMSE (W)	MAE (W)	RMSE (W)	MAE (W)	RMSE (W)
LR	59.48	75.84	29.32	106.66	715.76	975.67
DTR	50.94	71.39	48.64	151.65	363.36	782.62
LSTM	25.74	58.82	10.99	36.59	300.04	735.07
AEFLSTM	14.4	50.6	4.89	28.33	232.37	668.01

## Data Availability

Not applicable.

## Abbreviations

The following abbreviations are used in this manuscript:

CNN	Convolutional neural network
DR	Demand response
DTR	Decision tree regression
DWT	Discrete wavelet transform
FFT	Fast Fourier transform
HEMS	Home energy management system
HMM	Hidden Markov model
ILM	Intrusive load monitoring
KNN	k-nearest neighbors
LR	Linear regression
LSTM	Long short-term memory
LPF	Low pass filter
MAE	Mean absolute error
NILM	Non-intrusive load monitoring
RMSE	Root mean square error
RNN	Recurrent neural network
SD	Seasonal decomposition
SVM	Support vector method
